# Dysregulation of the endocannabinoid system – a key factor in the progression of multiple sclerosis?

**DOI:** 10.25122/jml-2025-0146

**Published:** 2025-09

**Authors:** Andreea-Cristina Paraschiv, Cristiana Văcăraș, Cristian Marge, Vitalie Văcăraș

**Affiliations:** 1 Department of Neurology and Pediatric Neurology, Iuliu Hațieganu University of Medicine and Pharmacy, Cluj-Napoca, Romania; 2 Pelican Medical Center Oradea, Faculty of Medicine, Oradea, Romania

**Keywords:** multiple sclerosis, anandamide, 2-arachidonoylglycerol, endocannabinoid system, teriflunomide therapy, disease progression, neuroinflammation, MS, Multiple sclerosis, AEA, Anandamide, 2-AG, 2-arachidonoylglycerol, RRMS, Relapsing-remitting multiple sclerosis, EDSS, Expanded Disability Status Scale, MMSE, Mini-Mental State Examination, SF-36, 36-item Short Form Health Survey (quality of life questionnaire), ANOVA, Analysis of variance, CNS, Central nervous system, SPMS, Secondary progressive multiple sclerosis, OCT, Optical coherence tomography, ECS, Endocannabinoid system, TNF-α, Tumor necrosis factor alpha, IL-1β, Interleukin 1 beta, IL-6, Interleukin 6, IL-8, Interleukin 8, CB1, Cannabinoid receptor type 1, CB2, Cannabinoid receptor type 2, FAAH, Fatty acid amide hydrolase, MAGL, Monoacylglycerol lipase, SOD1, Superoxide dismutase 1 (gene used in transgenic mouse models), CSF, Cerebrospinal fluid, MRI, Magnetic resonance imaging, CBD, Cannabidiol, BMI, Body mass index, ELISA, Enzyme-linked immunosorbent assay, LQ, Life quality, BDNF, Brain-derived neurotrophic factor, TrkB, Tropomyosin receptor kinase B

## Abstract

The endocannabinoid system has been implicated in the pathophysiology of multiple sclerosis (MS), yet its role across different disease stages and under disease-modifying treatment remains incompletely understood. This study aimed to evaluate plasma levels of anandamide (AEA) and 2-arachidonoylglycerol (2-AG) in patients with MS at different clinical stages, and to explore their associations with disability, cognition, and quality of life, as well as the potential influence of teriflunomide therapy. Thirty participants were enrolled: ten healthy controls, ten newly diagnosed relapsing–remitting MS (RRMS) patients in acute relapse, and ten teriflunomide-treated RRMS patients in remission. Plasma AEA and 2-AG were measured by ELISA; clinical assessments included the Mini-Mental State Examination (MMSE) and the SF-36 quality-of-life questionnaire. No significant group differences were observed overall in 2-AG (*P* > 0.05). AEA showed a non-significant overall group effect (ANOVA, *P* = 0.0919) with a trend toward lower AEA in newly diagnosed patients compared to healthy controls (mean difference = −5.95 ng/ml, SE = 2.66; *P* = 0.098). In the teriflunomide group, AEA and 2-AG were strongly positively correlated (r = 0.882, *P* < 0.001). Additionally, SF-36 scores were positively associated with MMSE (r = 0.706, *P* = 0.023). Furthermore, SF-36 total scores were significantly lower in newly diagnosed patients compared to controls (post-hoc *P* = 0.044). These findings suggest possible early dysregulation of the endocannabinoid system in MS and indicate that teriflunomide treatment is associated with a strengthened AEA–2-AG relationship. Larger, longitudinal studies are warranted to confirm these observations and to assess clinical implications for disease progression and patient quality of life.

## Introduction

Multiple sclerosis (MS) is a chronic neurodegenerative autoimmune disease of the central nervous system (CNS) that affects more than 2.3 million people worldwide, significantly reducing their quality of life [1]. This decline is attributed to clinical relapses and persistent symptoms, such as spasticity and pain. In 85% of cases, the diagnosis is established following the occurrence of an isolated clinical syndrome. The most common form of MS is relapsing-remitting multiple sclerosis (RRMS), accounting for 80–85% of all cases, which frequently progresses over time to secondary progressive multiple sclerosis (SPMS).

Several effective tools have been developed to predict the progression of the disease, including the Expanded Disability Status Scale (EDSS), the number of T2-hyperintense lesions, grey matter damage, optical coherence tomography (OCT), and serum neurofilament light chain levels [2]. Patient-reported outcome measures, such as the 36-Item Short Form Survey (SF-36), capture subjective experiences of symptoms and quality of life, but are limited by individual perception. Cognitive function is often assessed using the Mini-Mental State Examination (MMSE), which provides a rapid global evaluation but may lack sensitivity in detecting subtle deficits in processing speed and executive function that are characteristic of MS.

The endocannabinoid system (ECS) plays a critical role in the pathophysiology of MS by promoting neuroprotection, encouraging cell proliferation, and modulating the immune response. Endocannabinoids suppress the release of pro-inflammatory cytokines, including tumor necrosis factor alpha (TNF-α) and interleukins (IL-1β, IL-6, and IL-8), while stimulating nitric oxide production [3]. Anandamide (AEA) and 2-arachidonoylglycerol (2-AG), two key endocannabinoids, activate cannabinoid receptors CB1 and CB2. Their hydrolysis is catalyzed by the enzymes fatty acid amide hydrolase (FAAH) and monoacylglycerol lipase (MAGL), respectively. In superoxide dismutase 1 (SOD1) transgenic mouse models, elevated levels of AEA and 2-AG have been observed in the lumbar spinal cord, and pharmacological inhibition of MAGL has been shown to attenuate disease progression [4].

In human studies, conflicting findings have been reported. SPMS patients exhibit elevated plasma levels of AEA compared to controls, with no significant differences in 2-AG concentrations [5]. Conversely, increased cerebrospinal fluid (CSF) levels of AEA have been observed in patients with contrast-enhanced MRI lesions, independent of lesion volume [6]. However, no studies have compared endocannabinoid plasma levels between newly diagnosed MS patients and relapse-free RRMS patients undergoing teriflunomide treatment.

This study aimed to investigate the relationship between blood levels of AEA and 2-AG and key clinical parameters, such as disability and quality of life, in MS patients. Our findings may help clarify the role of ECS in the onset and progression of MS, providing a basis for identifying predictive biomarkers. Furthermore, cannabis-based treatments, including nabiximols (Δ9-tetrahydrocannabinol) and cannabidiol (CBD), have shown symptom relief in self-medicating patients [1] and are approved for clinical use in certain countries [4]. However, MAGL and FAAH inhibitors may represent more promising therapeutic options, as they lack the psychoactive effects of cannabis. This study also seeks to identify patient subgroups that may benefit from the controlled administration of these inhibitors.

## Material and Methods

### Study design and participants

This was a prospective, observational pilot study involving 30 participants recruited from the Neurology Department of Cluj-Napoca County Hospital, Romania. Participants were divided into three groups: healthy controls (*n* = 10; age range 23–41, mean age 28.2), newly diagnosed treatment-naive relapsing–remitting multiple sclerosis (RRMS) patients in acute relapse (*n* = 10; age range 22–61, mean age 35.9), and RRMS patients in clinical remission while receiving teriflunomide therapy (*n* = 10; age range 26–54, mean age 39.2). All subjects were Caucasian (18 women, 12 men; overall age range, 23–61; mean age, 34.43).

Treatment-naive RRMS patients were diagnosed with relapsing-remitting multiple sclerosis using the McDonald criteria and had not received any treatment at the time of blood collection. Treated RRMS patients were undergoing treatment with teriflunomide (Aubagio) and had experienced a relapse-free period of at least three months (clinical remission, age range 26–54, mean age 39.2). Patients and volunteers with abnormal BMI, a history of cardiovascular events, inflammatory bowel disease, or other autoimmune conditions were excluded from the study. None of the participants reported the use of exogenous cannabinoids or cannabis-based medications. All participants signed informed consent, and the study was approved by the Ethical Committee of the University of Medicine and Pharmacy ‘Iuliu Hatieganu,’ Cluj-Napoca, on September 28, 2020.

### Clinical and cognitive assessments

Disability was evaluated with the EDSS. Global cognitive screening was performed using the MMSE. Health-related quality of life was measured using the Orthotoolkit SF-36 Summary Score (SF-36). To ensure consistency, all evaluations were conducted by the same examiner.

### Blood sampling and biochemical assays

Venous blood samples were collected into EDTA tubes, centrifuged, and properly stored. Plasma concentrations of AEA and 2-AG were quantified using commercially available ELISA kits produced by MyBioSource, and results were calculated using a standard curve. Laboratory personnel from the Pharmacology Department of the University of Medicine and Pharmacy Iuliu Hatieganu were blinded to clinical group allocation.

### Determination of AEA

Specimens were first examined for signs of hemolysis. Reagents were brought to room temperature. The standard solution (12 ng/mL) was serially diluted to prepare a set of standards (6 ng/mL, 3 ng/mL, 1.5 ng/mL, 0.75 ng/mL). Fifty microliters (µl) of the standard solution were added to the standard wells, and 40 µl of the sample was added to the sample wells. Subsequently, 10 µl of anti-AEA antibody and 50 µl of streptavidin-HRP were added to each well, including the standard wells. The plate was incubated for 60 minutes at 37°C, then washed five times with wash buffer. Afterward, 50 µl of substrate solution A and 50 µl of substrate solution B were added to each well, followed by a 10-minute incubation at 37°C in the dark. Finally, 50 µL of the stop solution was added, and the optical density was measured using a microplate reader at 450 nm.

### Determination of 2-AG

Reagents were brought to room temperature. The standard solution (300 ng/mL) was diluted to prepare a set of standards (100 ng/mL, 33.33 ng/mL, 11.11 ng/mL, 3.7 ng/mL). Fifty microliters (µl) of the standard, blank, or sample was added to the respective wells, followed by 50 µl of reactant detector A. The plate was shaken and incubated for 60 minutes at 37°C, after which 350 µl of wash buffer was added. One hundred microliters (µl) of reactant detector B was added, and the plate was incubated at 37°C for an additional 30 minutes, followed by five washes. Ninety microliters of substrate solution were added to each well, and the plate was incubated for 10 minutes at 37°C in the dark. Finally, 50 µL of the stop solution was added, and the optical density was measured using a microplate reader at 450 nm.

### Statistical analysis

All statistical analyses were performed using SPSS version 25 (IBM Corp.). Continuous variables are presented as means ± standard deviations (SD), and categorical variables are presented as counts and percentages. The primary hypotheses were that plasma AEA and 2-AG concentrations would differ across the three study groups (healthy controls, newly diagnosed treatment-naive RRMS, and teriflunomide-treated RRMS) and that circulating endocannabinoid concentrations would be associated with clinical measures (EDSS), cognitive screening (MMSE), and health-related quality of life (SF-36). Prior to inferential testing, the distributional properties of continuous variables were examined using the Shapiro–Wilk test and Q–Q plots, and homogeneity of variances was assessed by Levene’s test. Where distributional assumptions were not met for secondary or exploratory analyses, nonparametric approaches were considered. The principal between-group comparisons reported in the results section employed parametric methods because assumptions were adequately satisfied for those variables.

Between-group comparisons for continuous outcomes were conducted with one-way analysis of variance (ANOVA). Post-hoc pairwise comparisons were adjusted for multiple testing using the Sidak correction. Bivariate associations between continuous measures were evaluated with Pearson correlation coefficients. All inferential tests were two-sided, and a nominal significance threshold of α = 0.05 was used. Exact *P* values are reported throughout the results section (with *P* < 0.001 reported as *P* < 0.001). Ninety-five percent confidence intervals (95% CIs) for mean differences are provided where available. Effect sizes are reported for the primary analyses: partial eta squared (partial η^2^) is presented for ANOVA results, and Pearson’s r is reported for correlations. Where pairwise mean differences are quoted from post-hoc output, the originally produced standard errors (SE) are reported verbatim and explicitly labelled as SE; descriptive dispersion in tables is given as mean ± SD in accordance with SAMPL reporting recommendations.

Because this investigation was conducted as a pilot study, no a priori sample size calculation was performed; recruitment was based on feasibility and case availability. To assist interpretation of non-significant and borderline results, post-hoc power estimates were computed from the observed test statistics using standard noncentrality-parameter methods. For example, the ANOVA for AEA yielded F(2,27) = 2.625 (*P* = 0.0919), corresponding to partial η^2^ = 0.163 and Cohen’s f ≈ 0.44, and the post-hoc power to detect that observed effect with N = 30 (*n* = 10 per group) was approximately 0.52. By contrast, the ANOVA for 2-AG produced F(2,27) = 0.114 (*P* = 0.893), partial η^2^ ≈ 0.008, and Cohen’s f ≈ 0.09, with post-hoc power ≈ 0.07. The SF-36 comparison (F(2,27) = 3.665, *P* = 0.039) corresponded to partial η^2^ = 0.214 (Cohen’s f ≈ 0.52) and post-hoc power ≈ 0.67, whereas the EDSS comparison (F(2,27) = 18.62, *P* < 0.001) reflected a large effect (partial η^2^ = 0.580; Cohen’s f ≈ 1.17) with power effectively ≈1.00. Within-group correlation analyses in the teriflunomide group (*n* = 10) showed r = 0.882 for AEA versus 2-AG (*P* < 0.001; post-hoc power ≈ 0.996) and r = 0.706 for MMSE versus SF-36 (*P* = 0.023; post-hoc power ≈ 0.70). Readers are referred to Supplementary Table S1 for a tabulated summary of test statistics, derived effect sizes, and post-hoc power estimates.

The pilot nature and small, convenience sampling strategy of the present study limit sensitivity to detect small-to-moderate effects; therefore, non-significant findings are interpreted conservatively. To inform planning of definitive studies, approximate total sample sizes required for 80% power at α = 0.05 in a balanced three-group ANOVA are as follows: for Cohen’s f = 0.25 (small–medium) *n* ≈ 157 (≈52 per group), for Cohen’s f = 0.40 (medium) *n* ≈ 63 (≈21 per group), and for Cohen’s f = 0.50 (large) *n* ≈ 42 (≈14 per group).

## Results

Participant demographic and baseline clinical characteristics were described previously in the Material and Methods section.

**Figure 1:**
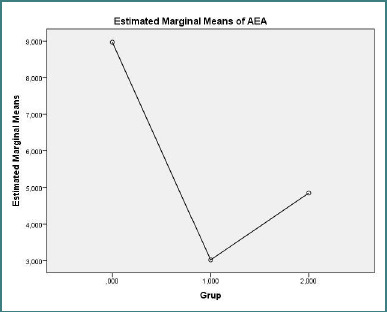
Estimated marginal mean plasma AEA (ng/mL^-1^) for control group (*n* = 10), treatment-naive RRMS (*n* = 10), and treated RRMS (*n* = 10). **One-way ANOVA F(2,27) = 2.625, *P* = 0.0919**.

**Figure 2:**
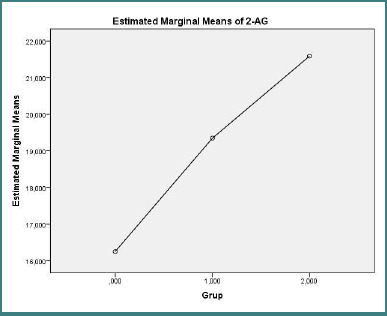
Estimated marginal mean plasma 2-AG (ng/mL^-1^) for control group (*n* = 10), treatment-naive RRMS (*n* = 10), and treated RRMS (*n* = 10). **One-way ANOVA: F(2,27) = 0.114, *P* = 0.893**.

Plasma endocannabinoid concentrations are presented in Figure 1 (AEA) and Figure 2 (2-AG). One-way ANOVA indicated no significant overall group difference for 2-AG (F(2,27) = 0.114, *P* = 0.893). For AEA, the overall ANOVA approached but did not reach statistical significance (F(2,27) = 2.625, *P* = 0.0919); post-hoc pairwise comparisons revealed a trend toward lower AEA concentrations in the treatment-naive RRMS group compared with healthy controls (mean difference = −5.95 ng/ml, SE = 2.66; *P* = 0.098). No significant differences in AEA were observed between the treated RRMS group and control group (*P* = 0.348) or between the treatment-naive RRMS group and treated RRMS patients (*P* = 0.873).

**Figure 3:**
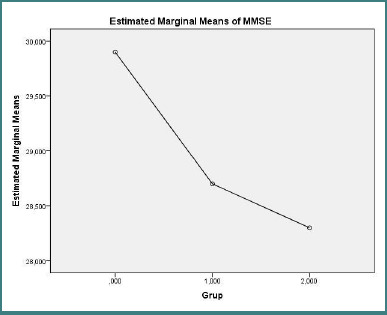
Estimated marginal mean MMSE (points) for control group (*n* = 10), treatment-naive RRMS (*n* = 10), and treated RRMS (*n* = 10). **One-way ANOVA F(2,27) = 2.011, *P* = 0.154**.

**Figure 4:**
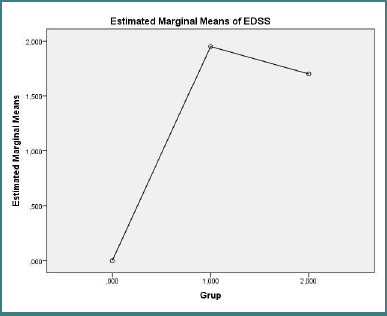
Estimated marginal mean EDSS for control group (*n* = 10), treatment-naive RRMS (*n* = 10), and treated RRMS (*n* = 10). **One-way ANOVA F(2,27) = 18.62, *P* < 0.001; control vs patient groups, P < 0.001**.

Cognitive function and disability outcomes are reported in Figure 3 (MMSE) and Figure 4 (EDSS). There were no significant differences in MMSE scores across the three groups (one-way ANOVA, F(2, 27) = 2.011, *P* = 0.154). By contrast, EDSS scores differed markedly between groups (one-way ANOVA F(2,27) = 18.620, *P* < 0.001), with post-hoc tests indicating that healthy controls had significantly lower disability scores than both treatment-naive and treated RRMS patients (control vs treatment-naive RRMS: mean difference = −1.950, SE = 0.348, *P* < 0.001; control vs. treated RRMS: mean difference = −1.700, SE = 0.348, *P* < 0.001).

**Figure 5:**
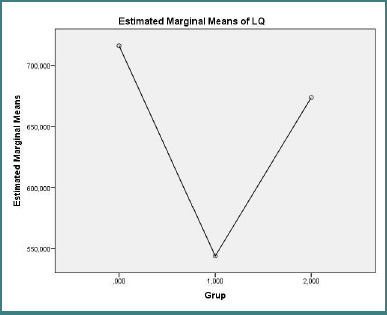
Estimated marginal mean SF-36 total score (model-derived) for control group (*n* = 10), treatment-naive RRMS (*n* = 10), and treated RRMS (*n* = 10). **One-way ANOVA F(2,27) = 3.665, *P* = 0.039; control vs treatment-naive post-hoc, *P* = 0.044**.

Health-related quality of life, assessed by SF-36 total scores (Figure 5), differed significantly between groups (one-way ANOVA F(2,27) = 3.665, *P* = 0.039). Post-hoc pairwise comparisons showed that healthy controls reported higher SF-36 total scores compared with treatment-naive RRMS patients (post-hoc *P* = 0.044); differences between treated patients and controls were not statistically significant.

In the treated RRMS group, AEA and 2-AG concentrations were strongly positively correlated (Pearson’s r = 0.882, *P* < 0.001). Additionally, MMSE scores were positively associated with SF-36 total scores in the treated RRMS group (Pearson’s r = 0.706, *P* = 0.023). No other correlations reached statistical significance after correction for multiple comparisons.

The original post-hoc output provided standard errors for mean differences; these SE values are reported verbatim in the text and explicitly labeled as SE. A complete tabulation of means, SDs, test statistics, effect sizes, and post-hoc power estimates is provided in Supplementary Table S1.

## Discussion

Our study offers further evidence of endocannabinoid system dysregulation in multiple sclerosis. The observed trend toward decreased AEA levels in newly diagnosed, untreated RRMS patients, while not statistically significant in our limited sample, aligns with previous findings in animal models and CSF studies [7,8]. This suggests a potential role for AEA in the early stages of MS pathogenesis. However, larger studies are needed to confirm this observation.

The significant positive correlation between AEA and 2-AG levels in patients treated with teriflunomide is noteworthy. This suggests that teriflunomide may influence the balance of these endocannabinoids, potentially contributing to its therapeutic mechanism. Further research should explore this interaction.

The discrepancy between our findings and previous reports regarding 2-AG levels [5,8] may be due to methodological differences, such as the use of ELISA in our study compared to spectrometry in others. Alternatively, 2-AG levels might indeed decrease after an acute MS attack, but this effect was not statistically significant in our smaller sample. Further investigation is needed to resolve this discrepancy.

The observation of considerable heterogeneity in endocannabinoid levels among healthy controls suggests that other, as yet unidentified factors may influence ECS regulation. These factors could include lifestyle factors, genetic predispositions, or subclinical inflammatory processes. Emerging research highlights the importance of the gut microbiota in modulating immune responses and potentially influencing MS. Alterations in gut microbiota composition and function have been linked to disease activity and progression in MS [9]. It is possible that the observed heterogeneity in our control group could be related to variations in gut microbiota profiles. Future studies should explore these factors, including the gut microbiome, to provide a clearer understanding of the regulation of the ECS. This is particularly relevant given that MS therapies themselves can impact the gut microbiota, potentially influencing treatment response [10]. The heterogeneity in the control group, along with our limited sample size, represents a limitation of our study.

The lack of significant differences in MMSE scores across groups suggests that overt cognitive impairment was not a prominent feature in our sample. However, the significantly higher EDSS scores in newly diagnosed RRMS patients are consistent with early neurological deficits. This underscores the need for more effective therapies to prevent the accumulation of disability in MS. While teriflunomide appears to have a positive influence on the disease course, as evidenced by the relative preservation of cognitive function in treated patients, further research is warranted. Furthermore, while current MS therapies primarily target inflammation, there is growing interest in neuroprotective strategies. Brain-derived neurotrophic factor (BDNF), a key neurotrophin, plays a crucial role in neuronal survival and plasticity. Studies have investigated the effects of MS treatments on BDNF levels, suggesting potential neuroprotective mechanisms. For instance, glatiramer acetate, another common MS treatment, has been shown to influence BDNF and its receptor TrkB levels in RRMS patients [10], suggesting a potential role in neuroprotection. Future studies should also investigate the relationship between endocannabinoid levels and neurotrophic factors, such as BDNF, in MS, as well as the potential impact of teriflunomide on BDNF levels.

The observed decline in quality of life in newly diagnosed MS patients, likely due to physical symptoms and emotional distress following diagnosis, highlights the psychological burden of MS. The correlation between decreased quality of life and cognitive deficits is consistent with previous findings [11]. While we did not find a significant correlation between EDSS and quality of life, this may be due to the relatively short disease duration and the younger age of our patients. Future studies using more MS-specific quality of life measures may reveal a stronger association. The positive correlation between quality of life and cognitive function in treated patients suggests that preserving cognitive function is crucial for maintaining quality of life in MS.

Our findings demonstrate that the endocannabinoid system is dysregulated in MS, with a tendency for decreased levels of AEA and 2-AG following a relapse. This pattern was not observed in patients undergoing one year of treatment with teriflunomide, suggesting the therapeutic effectiveness of this drug in stabilizing endocannabinoid levels. However, studies involving larger participant numbers are needed to confirm these results and increase statistical power.

A positive correlation between AEA and 2-AG plasma levels was found in patients receiving immunomodulatory treatment, further indicating a systemic dysregulation of the ECS in MS. Additionally, life quality in MS patients is notably lower after diagnosis compared to healthy controls, with quality of life tending to decrease in parallel with cognitive deficits in treated patients. The physical symptoms measured by the EDSS had a smaller impact on quality of life compared to cognitive impairment.

Further research is essential to evaluate the internal and external factors that influence the endocannabinoid system in MS. These factors should be taken into account in future studies to better understand the complexities of ECS regulation and its potential therapeutic implications.

## Conclusion

In this pilot study of 30 participants (ten per group), we observed patterns consistent with early dysregulation of the endocannabinoid system in multiple sclerosis. Although the omnibus ANOVA for plasma AEA did not reach conventional significance (F(2,27) = 2.625, *P* = 0.0919), pairwise comparisons indicated a trend toward lower AEA in newly diagnosed, treatment-naive RRMS patients compared with healthy controls (mean difference = −5.95 ng•mL^-1^, SE = 2.66; *P* = 0.098). No significant group differences were detected for 2-AG (F(2,27) = 0.114, *P* = 0.893). In the teriflunomide-treated cohort, AEA and 2-AG were strongly positively correlated (r = 0.882, *P* < 0.001). Additionally, cognitive performance (as measured by MMSE) correlated with health-related quality of life (as assessed by SF-36) in the treated group (r = 0.706, *P* = 0.023). Disability (as measured by EDSS scores) differed markedly across groups (F(2,27) = 18.62, *P* < 0.001), and SF-36 scores were lower in newly diagnosed patients compared to controls (post-hoc *P* = 0.044).

Taken together, these findings are compatible with the hypothesis that perturbations in endocannabinoid signaling accompany early MS and that immunomodulatory therapy with teriflunomide may be associated with a partial normalization or re-coupling of AEA and 2-AG dynamics. However, given the pilot nature of the study, its small sample size (post-hoc power for the observed AEA effect ≈ 0.52), and the cross-sectional design, causal inferences are not warranted. Methodological considerations — including the use of ELISA rather than mass spectrometry for endocannabinoid quantification, possible unmeasured confounders (such as lifestyle, diet, comorbidities, and gut microbiota), and reliance on a brief cognitive screen (MMSE) — further temper definitive conclusions.

Future work should validate these preliminary observations in larger, longitudinal cohorts with pre-specified sample-size calculations, employ more sensitive assays (e.g., LC–MS/MS) and comprehensive cognitive batteries, and integrate complementary biological measures (CSF endocannabinoid profiling, neurofilament light, BDNF, and microbiome analyses). Such studies should also evaluate the temporal relationship between disease activity, treatment exposure, and endocannabinoid dynamics and explore whether modulating endocannabinoid metabolism (for example, via MAGL or FAAH inhibition) has measurable clinical or neuroprotective benefits. If confirmed, circulating endocannabinoids could become useful adjunct biomarkers to stratify patients and to monitor therapeutic effects in MS.

In sum, our results support a role for the endocannabinoid system in MS pathophysiology and highlight teriflunomide-associated changes in endocannabinoid relationships as an avenue worthy of further, adequately powered investigation.

## Supplementary Material



## Data Availability

Due to privacy concerns, the data and other information from this study are only available upon request from the study’s corresponding author.
