# Supplementary material for: Dysregulation of the endocannabinoid system – a key factor in the progression of multiple sclerosis?
Source: J Med Life. 2025 Sep;18(9):863–8. doi: 10.25122/jml-2025-0146 (PMC12577783; doi:10.25122/jml-2025-0146)
Supplement: Supplementary file 1 [file JMedLife-18-863-s001.pdf]

Supplementary Table 1. Effect sizes and post-hoc power estimates. The table below summarizes the reported test statistics, calculated effect sizes (partial η^2 and Cohen's f for ANOVA where applicable), and post-hoc power estimates (α = 0.05) based on the observed statistics and sample size N = 30 (n = 10 per group). Correlation power estimates are based on group-specific sample sizes where indicated.

Outcome	Test	Statistic	*P* value	partial η^2	Cohen's f	Post-hoc power (α=0.05)	Notes
AEA (plasma)	One-way ANOVA	F(2,27) = 2.625	0.0919	0.163	0.441	0.52	Trend toward lower AEA in treatment-naive vs controls (pairwise p = 0.098)
2-AG (plasma)	One-way ANOVA	F(2,27) = 0.114	0.893	0.008	0.092	0.07	No group differences
SF-36 (quality of life)	One-way ANOVA	F(2,27) = 3.665	0.039	0.214	0.521	0.67	Control vs newly diagnosed: post-hoc p = 0.044
EDSS (disability)	One-way ANOVA	F(2,27) = 18.620	< 0.001	0.580	1.17	≈1.00	Large effect; controls lower than RRMS groups
MMSE (cognition)	One-way ANOVA	F(2,27) = 2.011	0.154	0.130	0.386	0.42	No significant differences
AEA vs 2-AG (teri-flunomide group)	Pearson correlation	r = 0.882 (n=10)	< 0.001			0.996	Strong positive correlation within treated group
MMSE vs SF-36 (ter-iflunomide group)	Pearson correlation	r = 0.706 (n=10)	0.023			0.70	Moderate positive correlation within treated group

Notes: Partial η^2 values were computed from reported ANOVA F statistics using partial η^2 = (F × df_effect) / (F × df_effect + df_error). Cohen's f was derived from partial η^2 via f = sqrt(η^2 / (1 − η^2)). Post-hoc power estimates were obtained using standard F-test and *t*-test noncentrality parameter approaches based on the observed statistics.

© 2025 by the authors. This article is an open access article distributed under the terms and conditions of the Creative Commons Attribution (CC BY 4.0) license.
